# The Interplay between Socioeconomic Status, Parenting and Temperament Predicts Inhibitory Control at Two Years of Age

**DOI:** 10.3390/children10061085

**Published:** 2023-06-20

**Authors:** Ángela Conejero, M. Rosario Rueda

**Affiliations:** 1 Department of Developmental and Educational Psychology, University of Granada, 18071 Granada, Spain; 2 Mind, Brain and Behavior Research Center (CIMCYC), University of Granada, 18071 Granada, Spain; 3 Department of Experimental Psychology, University of Granada, 18071 Granada, Spain

**Keywords:** inhibitory control, self-regulation, toddlers, socioeconomic status, parenting, temperament, effortful control

## Abstract

This paper investigates the interplay between environmental factors (socioeconomic status (SES) and parenting) and temperament in the development of inhibitory control (IC) at 2 years of age. We administered to toddlers (*n* = 59) a delay of gratification task which measures IC in the context of self-regulation. Parents reported their toddlers’ temperament, parenting strategies, and SES. We hypothesized that poorer IC would be associated with more reactive temperament, less effortful control, lower SES and inconsistent/coercive parenting practices. Finally, we explored the interaction between temperament, parenting and SES. We found that both coercive parenting and low-SES were negatively correlated to IC at the age of 2 years. Temperamental reactivity was unrelated to IC, whereas temperamental effortful control (EC) was positively associated with IC. Results revealed a moderation effect of EC on the influence of coercive parenting and SES in toddlers’ IC. Toddlers from lower SES backgrounds and with lower EC were more affected by inconsistent/coercive parenting practices and showed the poorest IC. In contrast, toddlers exhibiting high and average levels of EC seemed to be protected from the detrimental effect of low-SES and inconsistent/coercive parenting on IC. These results suggest that strengthening toddlers’ EC and improving parents’ parenting skills might be especially relevant for the development of IC in the context of self-regulation, particularly by preventing self-regulatory problems in children from socioeconomically deprived environments. Future studies with larger samples, focusing on populations from severe socioeconomically deprived environments, or intervention studies will be needed in order to confirm and expand our findings.

## 1. Introduction

From the very moment a child is born, they face the challenge of interacting with an overstimulating and constantly changing world. Success in this endeavour will mainly depend on children’s inhibitory control (IC). IC is considered one of the three main components of executive functions [1,2] and entails suppressing automatic or prepotent responses, ignoring irrelevant information or restraining immediate impulses in order to achieve one’s desired outcomes. Thus, IC prevents children reacting to immediate and momentary events and facilitates goal-directed behaviour [3]. The ability to voluntary control our behaviour in everyday life situations, known as self-regulation, is highly determined by IC skills [4,5]. IC is fundamental for dealing with temptations, modulating emotional reactions, overriding habits and acting thoughtfully instead of behaving on impulse. Recent theoretical models explaining early self-regulation skills in children integrate IC at the core of the development of self-regulation [6,7]. In fact, the development of IC has a crucial role in fostering self-regulation, which in turn has a great impact on children’s academic learning and psychosocial adjustment [8,9,10]. The influence of early individual differences in IC on the development of self-regulatory skills can extend far beyond childhood into adolescence and adulthood. Children’s IC at the age of three can predict psychosocial maladjustment in adulthood. Children who failed to control their impulse of eating a delectable snack had an increased probability of becoming involved in delinquency, substance abuse or gambling as adolescents and adults [11,12]. Some evidence further indicates that deficiencies in IC are on the basis of the self-regulatory problems typically observed in developmental disorders such as attention deficit disorder or autism [13,14].

Although there is a growing body of literature on the development of IC and self-regulation, studies have primarily focused on children from preschool ages onwards and there is still little published data comprising the period before the third year of life [15]. However, developmental research has started to demonstrate the remarkable change in toddlers’ IC as they progressively make more evident attempts to self-regulate. The characteristic stubbornness of toddlers leads to inaccurate thinking that children at this age are less competent at controlling their behaviour and feelings than they really are. Indeed, 2-year-olds start to develop active strategies that help them to increase the effectiveness of self-regulation [16]. More evidence in recent years has suggested that, precisely from toddlerhood there is a clear improvement in IC and self-regulatory capacities, which will continue to noticeably grow during the following early childhood years [17,18,19]. Indeed, a faster growth of IC skills is observed in toddlerhood, with a steadier increase during the later stage of early childhood [20].

Tasks measuring IC of children in the context of behaviour self-regulation often include some rewards or appetitive stimuli to elicit the desired level of excitement and motivation in children [21]. Examples of such tasks are the so-called Delay of Gratification task [22] or the Snack Delay task [23], in which children have to resist eating a treat placed at a reaching distance. These kinds of tasks are self-regulatory; they involve inhibiting a highly desired response to comply with an instruction, engaging IC [24]. Studies using such simple self-regulatory tasks have generally observed individual differences among toddlers in successfully inhibiting their behaviour. Prior studies have reported moderate-to-high variability in the performance of two-year-olds in this kind of delay tasks [23,25]. Moreover, it has been estimated that about 50% of toddlers between 2 and 3 years of age fail to delay, clearly challenging their IC [26]. All in all, this suggests that individual differences in IC that we observed in the general population from childhood throughout the life-span can already be seen from toddlerhood.

One remaining question is the contribution of different environmental and constitutional factors to the emergence of individual differences in IC. Literature investigating individual differences in executive functions in children mainly focused on the impact of environmental factors such as socioeconomic status (SES) or parenting. Overall, these studies find that executive functions (including IC) are quite sensitive to environmental features. With regard to SES, children raised in families from lower-SES contexts generally show poorer performance in executive functions tasks [27,28], which is linked to reduced cortical thickness and lower white matter density in prefrontal brain structures underlying executive skills, such as the cingulate cortex [29]. Although there is cumulative evidence demonstrating that SES has an impact on the development of IC during childhood, much less is known about the toddlerhood period. There is some work that suggests that SES disparities relate to individual differences in IC as measured by delay tasks during the second and third year of life. For example, Lengua et al. [9] found that 3-year-olds from low SES backgrounds develop a poorer ability to delay gratification. According to their results, the IC of children from socioeconomically deprived environments is affected by a confluence of environmental variables such as low parental education, frequent residential changes, unstable family structure or a crowded household. Similarly, Lecheile et al. [30] reported an association between SES and IC at 30 months. However, this study indissociably combines children’s scores in a delay task and parents’ reported effortful control (EC; a temperament trait involving the tendency to self-regulate) in a general index of IC. More recently, Elliot et al. [31] conducted an online study where they recorded self-regulation behaviours exhibited by children during the testing procedure as a measure of IC. These behaviours included the ability to wait between experimental tasks and the impulsivity of their responses. Authors found a positive relation between IC and SES. Unlike the aforementioned studies, this research did not employ a delay task. Instead, it used indirect measures of the ability of children to delay, relaying in observers’ ratings of specific behaviours in a non-standardized situation. 

Concerning parenting, there is evidence that low-quality parenting could also have a negative impact on children’s general executive functioning from very early on. More specifically, the influence of parenting practices in the development of IC seems to be especially relevant in early years, a period of great growth in IC skills [32]. Inconsistent parenting strategies, low sensitivity to children’s needs and coercive parenting style have been related to poorer IC and externalizing behaviour problems [33,34,35]. Intrusive and directive parenting is particularly associated with poorer IC in delay tasks in children between 2 and 4 years of age [36,37]. In contrast, high-quality parenting could benefit early IC development. Children who demonstrated greater self-restraint in a delay of gratification task at the age of 4 years were those whose parents showed higher sensitivity to children’s needs during infancy and toddlerhood [38]. Parents’ responsiveness has been also associated with greater IC of 5-year-olds [39] and 2-year-olds [40] in delay tasks. However, literature on the impact of positive vs. negative parenting practices in toddlerhood offers some contradictory findings. According to Merz et al. [41] the use of directive language (a characteristic of coercive parenting style linked to intrusiveness) during mother–child interactions solely predicted toddlers’ IC, in contrast to mothers’ responsiveness. In light of some longitudinal research, both negative and positive parenting practices contribute to the development of IC during toddlerhood, but their effects vary at different ages. Once more, conflicting results exist regarding this matter. Moil et al. [18] proposed that coercive parenting practices may help to explain initial individual differences in IC in the first two years of age, whereas positive parenting would be related to the growth in IC skills across the following years. However, Geeraerts et al. [20] discovered the opposite pattern: positive parenting practices were explaining initial individual differences in IC during toddlerhood, while a deceleration in the growth rate of IC skills in the subsequent years could be attributed to the effect of negative parenting practices. A recent systematic review highlights that the relationship between parenting and the development of self-regulation could be unstable during the first years of life [42]. Authors suggest that children’s biological predispositions should be also considered when seeking to understand individual differences in self-regulatory skills, such as IC.

Building upon the idea that constitutional factors may also have an impact on the development of IC, a portion of research is focused on investigating the connection between individual differences in temperament and IC. Temperament refers to the observed differences in motor, emotional and attentional reactivity, together with the mechanisms involved in regulating such reactivity [43]. Temperament is considered a constitutional-based characteristic of children, which remains relatively stable throughout development, even from early years [44]. Individual differences in behavioural, emotional and attention reactivity can be observed from very early in development, so that parents can distinguish if their children are more or less irritable or active. It is generally found that these individual differences in temperament are also related to children’s executive functions. Higher levels of temperamental reactivity, either surgency/extraversion (SUR) or negative affect (NA), have been associated with poorer executive functioning [45,46,47]. Distinct temperamental profiles have been related to children’s differences in executive functions [48]. With regard to IC [45,46,47,48], higher probabilities of failing in delaying gratification are associated with higher activity levels and distress in two-year-olds [49]. However, EC is the temperament factor most closely related to inhibitory control. As already mentioned, EC refers to the predisposition for self-regulation in day-to-day situations, with IC proposed to be at the core of the development of this temperamental trait [50,51]. The ability to delay positively correlates to the IC scale, a component of the EC temperamental factor in the Child Behaviour Questionnaire, at the age of three [52].

Taken together, research to date recognizes the relevance of both environmental and constitutional aspects for understanding individual differences in IC. The bulk of research on early development of IC tends to focus on the effects of one or two of these variables (parenting, SES or temperament). Nevertheless, studies rarely examined the interplay between the three factors. Some studies have examined the interaction between temperament and SES in cognitive development. Ursache et al. [53] observed in a sample of low-income children that those with better general executive functioning at the age of 4 years also showed greater EC as toddlers. More attention has been paid to how SES and parenting interact. It has been suggested that environmental factors such as parenting may have a greater impact on the cognitive development of children from lower SES contexts in contrast to children raised in more advantageous contexts [54]. In other studies, parenting is proposed to mediate the impact of socioeconomic deprivation on cognitive development in children raised in impoverished environments [55], with some evidence indicating that the mother’s parenting style mediates the relation between SES and IC in young children [56]. However, the research on how the interplay between SES and parenting style shapes toddlers’ development of IC is still scarce, with no clear relation pattern currently established. In a study by Yu et al. [57], it was found that negative parenting practices did not mediate the impact of socioeconomic deprivation on children’s performance on a basic IC task before early childhood. This finding was observed in a sample of children from a low-SES background. Concerning toddlers’ ability to delay, results from Lengua et al. [58] suggest that both SES and parenting (specially negative parenting practices) contribute to explain 3-year-olds’ ability to delay. However, the interrelation between SES and parenting was not examined in this research. Likewise, Merz et al. [41] reported that the intrusiveness of mothers (as evidenced by the use of directive language in a parent–child interaction setting) predicted children’s ability to delay between the second and fourth year of life. In this study, no other variables apart from mother’s interaction style were taken into account. As far as we are concerned, no studies to date have addressed the mutual influence that SES, parenting and child temperament may have on individual differences in IC in toddlerhood.

In the present study, we investigated the early development of IC during the second year of life, considering the differential impact of environmental factors (SES and parenting strategies) and the individual differences in temperament. For that purpose, we measured IC with a delay task: the Snack Delay task [40]. We also asked parents to provide information about children’s temperament, parenting strategies and SES. In view of the reviewed literature, we expected: (1) higher SES as well as higher quality parenting will be related to better performance of toddlers in the Snack Delay task; (2) coercion and inconsistent parental practices would translate into poorer performance of toddlers in the Snack Delay task. Additionally, we explored whether child temperament moderated the relationship between SES, parenting and IC. Due to the insufficient evidence, no specific hypotheses were formulated regarding the moderation and interaction effects.

## 2. Materials and Methods

### 2.1. Participants

A total of 59 2-year-olds (26 male, 33 female) participated in the study (mean age = 26.62 months, SD = 0.90). Children were recruited by means of brochures distributed among nurseries in the city of Granada (Spain) and advertisements in local press and the University of Granada web bulletin board. To ensure that the sample represented the Granada population, the recruitment was carried out in neighbourhoods with diverse socioeconomic backgrounds. All children were born at term (>37 weeks of gestational age) and had no history of neurodevelopmental disorders. Parents or legal guardians provided informed consent for all children participating in the study. They received a 10 € gift voucher for educational toys in appreciation for their participation.

### 2.2. Procedure

Parents and children were greeted in the reception room by the experimenter where the study was explained and they were asked to sign the informed consent form. The experiment started after a warming-up period (5 min in which experimenter played with the child). Toddlers performed the Snack Delay task [40] among other experimental tasks not reported here, as they are not relevant for the purpose of this study. Children’s behaviour during the task was video recorded for offline coding. Parents completed a computerized web-based version of the temperament questionnaire and the parenting strategies scale at home within the week after their visit to the laboratory. SES information was obtained in a previous visit to the lab when the children were 16 months of age. The study was conducted in accordance with the Declaration of Helsinki. The procedures described here were approved by the Ethics Committee of the University of Granada.

#### 2.2.1. Temperament Assessment

Temperament was assessed with the Spanish short version of the Early Childhood Behaviour Questionnaire (ECBQ; [59]). Parents had to respond to 107 items on a 7-point scale, concerning how often they observed the described behaviour over the last week. This questionnaire measures three main temperament factors: surgency, negative affectivity and effortful control. The low intensity pleasure scale was excluded for obtaining the negative affectivity factor due to low reliability (Cronbach’s alpha < 0.6). Reponses from parents of 2 children were not received for this questionnaire.

#### 2.2.2. Socioeconomic Status Index

We used a custom-made survey to ask parents about three different aspects: parents’ education, parents’ occupation and family incomes. Parents reported their education level through a 7-point scale as follows: (1) No education; (2) Elementary school; (3) Secondary School; (4) High School; (5) Technical College/University diploma; (6) University Bachelor degree; and (7) Postgraduate studies. Parents also indicated their occupation status (unemployed or employed) specifying the sector, role, and type of contract if this was the case. Occupation was rated on a scale from 1 (unemployed) to 9 (manager) according to the Spanish Occupation Classification (CNO-11) from the Spanish National Institute of Statistics (BOE, 2010). Finally, we computed the income-to-need ratio. The total family incomes were divided by the poverty threshold incomes in Spain according to the data of the National Institute of Statistics of Spain (http://www.ine.es; accessed on 6 February 2014). A compound measure of SES was obtained by averaging the three measures after being transformed into z-scores.

#### 2.2.3. Parenting Measure

We used the Inventory of Parenting (IPC) [60] comprising 37 items to assess parenting strategies employed in the day-to-day interactions. Parents rated the usage of each strategy from 0 (never or almost never) to 3 (very often). The acceptatance and sensibility scale provided information about the use of strategies based on motivation, affect, sensitivity to children’s needs and reasoning (e.g., “When my son/daughter does something bad or something I don’t like, I explain to him/her what was wrong”). The inconsistency and coercion scale measured the use of strategies based on control or punishment, or the consistency in the use of the different strategies (e.g., “I threaten my son/daughter if he/she didn’t do what I asked for”). Cronbach’s alphas for both scales were 0.78 and 0.76, respectively. Parents of 2 children did not return their responses to this questionnaire.

#### 2.2.4. Snack Delay Task

Toddlers sat at a table on their parents’ lap, in front of the experimenter. Parents were instructed not to interfere and keep their interaction with their children at a minimum during the experiment. The experimenter placed a snack covered by a transparent plastic cup at a reaching distance from the toddler. A bell was placed at the side, next to the experimenter and visible to the toddler. Several runs with different waiting times (5, 10, 15 and 20 s) were conducted. Each trial started with asking the toddler to place their hands on a hand-shaped mat on the table, 15 cm away from the snack. After that, the experimenter asked the toddler not to take the snack until she rang the bell. A total of 4 trials with different waiting times (5, 10, 15 and 20 s) were administered. Children behaviour during the waiting time was coded as follows: 1 (ate the snack during the first half of the trial); 2 (ate the snack in the second half of the trial); 3 (touched the snack in the first half of the trial); 4 (touched the snack in the second half of the trial); 5 (touched the glass or the bell in the first half of the trial); 6 (touched the glass or the bell in the second half of the trial); 7 (the child waited until the end of the trial without touching the snack); 9 (waited until the end of the trial without moving their hands from the mat). Children who were not motivated by the snack were excluded from the analyses (*n* = 3). Children’s videotaped behaviour was coded by two trained research assistants who did not participate in administering the tasks and were blind to the main hypothesis of the study. About 30% of the videos were independently coded by both research assistants. The Krippendorff’s alpha test [61] was calculated to estimate the interrater reliability. The results showed a good interrater reliability (*α* = 0.86).

### 2.3. Analyses Plan

All analyses were run with IBM SPSS software, version 21. Given that the distribution of the studied variables did not significantly deviate from normality according to the Kolmogorov–Smirnoff test (all *p* > 0.05), parametric statistical analyses were performed. Pearson’s correlation analyses were carried out to explore the relationship between the main variable (performance of children in the Snack Delay task, measuring IC) and both temperamental and environmental factors. A 95% confidence interval level (CI) for the estimated correlation parameters was also computed. To test our hypothesis of a three-way interaction between SES, EC, and parenting style that explained individual differences in the Snack Delay task, we conducted a moderation analysis. Analysis was performed with the macro PROCESS for SPSS [62]. We estimated the coefficients at a confidence interval level of 95% using bias-corrected bootstrapping approach with 5000 samples. Since age showed no significant correlation with the performance of toddlers in the Snack Delay task (*r* = 0.16, *p* = 0.22), we did not include age as a covariate in our analyses for parsimony. Provided that sample size was not estimated a priori in this study (sample size was constricted by limited resources and time constraints) a sensitivity analysis [63] was performed in G Power [64] to determine whether the effect size of this analysis was sensitive enough to detect a moderation. The critical F value for the R^2^ increase in linear multiple regression (fixed model) with 80% power and α = 0.05 was calculated for our sample size.

## 3. Results

### 3.1. Descriptive statistics

The descriptive statistics are provided in Table 1. The mean, SD and valid sample are provided.

### 3.2. Correlation Analyses

As shown in Table 2, temperament, parenting and SES were related to IC as measured with the Snack Delay task. Regarding the temperament measures, the higher the score in the EC temperament scale, the greater the IC showed in the Snack Delay Task (*r* = 0.33, *p* < 0.05). However, the performance in the Snack Delay task was unrelated to toddlers’ SUR or NEG. In relation to parenting, only the coercion/inconsistency scale was correlated with the performance of toddlers in the Snack Delay task. Inconsistency in parenting practices and a coercive style was negatively associated with toddlers’ ability to delay (*r* = −0.25, *p* < 0.05). Finally, lower SES was associated with poorer performance in the Snack Delay task (*r* = 0.37, *p* < 0.01).

### 3.3. Moderation Analyses

**Figure 1:**
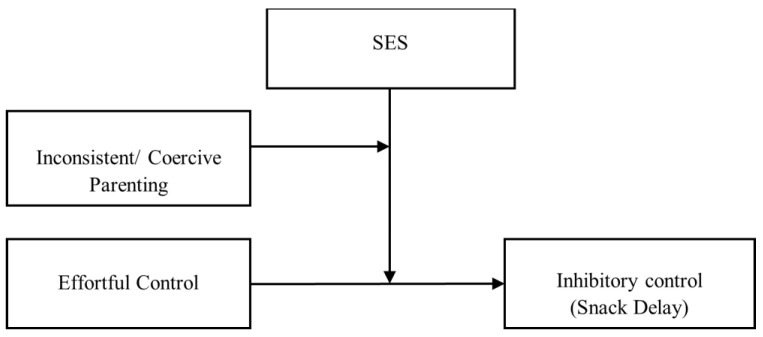
Interaction between SES, coercive/inconsistent parenting and toddlers’ EC to predict the performance of toddlers in the Snack Delay task (IC). Variables are depicted within boxes, while arrows illustrate the relationship between the predictors and the predicted variable.

**Figure 2:**
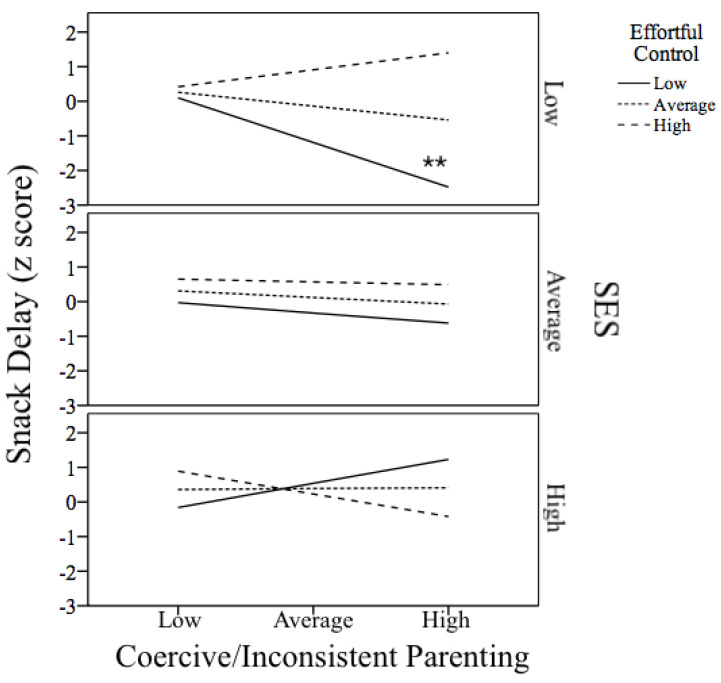
Interaction between SES, coercive/inconsistent parenting and toddlers’ EC to predict the performance of toddlers in the Snack Delay task (IC). Slopes predicting IC from inconsistency/coercive parenting are shown for Average, Low (below 1 SD) and High (above 1 SD) levels of EC and SES. ** *p* < 0.01.

We built our moderation model according to model number 3 (see Figure 1) following Hayes [62]. As shown in Figure 1, We tested whether the interaction between Inconsistent/Coercive Parenting, SES and Effortful Control predicted IC as measured with the Snack Delay task. Estimates with CIs for the whole model are available in Table S1. The general model was significant (*F*(7,38) = 4.11, *p* < 0.01, *R*^2^ = 0.43). Interaction between SES, EC and inconsistent/coercive parenting significantly predicted performance in the Snack Delay task. Adding the interaction term to the model significantly increased the proportion of explained variance (Δ*R*^2^ = 0.08, *F*(1,38) = 5.20, *p* < 0.05). Sensitivity analyses established the critical *F* value for the *R*^2^ increase into 3.19 for our sample size. As can be seen in Figure 2, performance in the Snack Delay task significantly decreased as a function of inconsistent/coercive parenting in the case of toddlers from a low-SES background that present low (from 1 SD below the mean) EC (*t*(38) = −3.11, *p* < 0.01). However, performance on the Snack Delay task was unrelated to parenting in the rest of the cases (*t*(38) < 1).

## 4. Discussion

The aim of this study was to examine individual differences in the IC of two-year-old toddlers. We aimed to explore the influence of temperament and environmental factors on individual variations in IC among toddlers, particularly focusing on their ability to delay gratification. Additionally, we investigated the interaction effect of these factors to better understand their joint contribution to IC. As we anticipated, IC was related to temperament and environmental variables such as parenting and SES. More specifically, our results demonstrated that the interplay between EC, SES and coercive/inconsistent parenting strategies predicted individual differences in two-years-olds’ ability to delay. According to our data, the IC of toddlers raised in lower SES contexts is especially affected by inconsistent/coercive parenting practices for those toddlers who also exhibited poorer EC. These children showed a decrease in the performance of the Snack Delay task when parents tended to be inconsistent in their parenting practices and had a coercive style.

As expected, IC was associated with parent-reported children’s temperament. However, not all temperament factors were related to the ability of toddlers to delay. Toddlers’ ability to delay was related to EC, but unrelated to temperamental reactivity (either NA or SUR). Our results support prior findings on the relationship between IC and EC in early years [40,65], but fail to demonstrate a relationship between IC and temperamental reactivity. A possible explanation could be that the performance in the Snack Delay task is not necessarily influenced by individual differences in temperamental reactivity, as this task does not intend to measure the intensity of an emotional reaction, but more specifically how children exert control over their impulses. In fact, measures such as the Snack Delay task may more easily elicit in children as young as two years of age the awareness of the need to control their impulses. Thus, this makes this task a sensitive measure of individual differences in IC in the context of self-regulation during this early stage of development. This is in accordance with the finding that performance in delay tasks is integrated into a common EC factor along with other self-regulation and cognitive control measures, while emotion-evoking tasks measuring variations in the intensity of children’s reactivity load into a separate factor [66].

Regarding environmental factors, both SES and parenting were related to toddlers’ IC in our data. As we hypothesised, lower SES and higher inconsistent parenting were related to toddlers’ poorer performance on the Snack Delay task. On the one hand, our research parallels previous findings indicating that children from lower SES backgrounds generally perform below high-SES children in delay tasks [9,30,31,65]. Socioeconomically deprived environments are usually described as more unpredictable and stressful [67,68], which might interfere with how toddlers learn about rewards, contingencies, and long-term outcomes. Apart from IC, delay tasks require toddlers to be able to follow basic instructions and comply with basic rules, which depends on how children build expectations about their environment. The lack of a predictable environment would make it harder for toddlers to create stable links between actions and consequences and, as a result, understand when and why they should control their behaviour. This would also lead to fewer opportunities for toddlers to put into practice the control of their behaviour. Alternatively, it has been suggested that socioeconomically disadvantaged families are limited in how much they can invest in educational materials and cognitively stimulating activities [69]. At the same time, the lower educational level of parents has been linked to a subestimation about the impact that stimulating children’s cognitive abilities may have on children`s development at early ages [70]. 

On the other hand, our results indicate that inconsistent and coercive parenting strategies may hinder the development of IC, which is essential to self-regulate in the Snack Delay task in order to refrain from their tendency to approach such a tempting snack. This is in agreement with some research showing that parents prone to be overly controlling of children’s behaviour may impair children’s development of the self-regulatory skills needed to succeed in delay of gratification tasks, including IC [71]. It has been suggested that coercion makes toddlers unable to autonomously regulate, limiting the number of experiences in which children actively control their own behaviour [72]. Children’s behaviour might be mainly regulated by parents with a coercive parenting style, limiting children’s experiences of implementing IC. Conversely, inconsistency may hinder toddlers from forming a stable reference on how they are expected to behave and the consequences of their behaviour [18,73]. Inconsistency in parenting practices is another element that contributes to make the environment less predictable, and can prevent children from establishing a clear association between actions and consequences, as we already mentioned. Unexpectedly, positive parenting practices (acceptatance and sensitivity to children’s needs) did not positively associate with individual differences in IC in our two-year-olds sample. Our findings align with previous research that has provided evidence of the prominence of negative parenting practices over positive parenting practices to explain individual differences in IC during toddlerhood [18,36,37,41,58]. The impact of negative and positive parenting practices on the development of IC may have different weights across development. According to our results and considering some cumulative evidence, the use of coercive and inconsistent practices by parents would affect children’s development especially during toddlerhood. This is consistent with some longitudinal data showing a differential effect of positive and negative parenting practices on the development of IC across childhood, depending on children’s age [41]. This pattern of association might be specific for the development of IC and other self-regulatory capacities, with parenting practices affecting the development of other cognitive skills in a different way across time. This could be potentially explored by new longitudinal studies. 

Finally, concerning the interplay between temperament and environmental factors, we found an interaction between parenting, SES and EC in predicting performance of toddlers in the Snack Delay task. Toddlers from low-SES backgrounds and poorer EC were particularly vulnerable to the impact of inconsistent/coercive parenting practices, showing a reduced IC in the Snack Delay task. In contrast, high and average EC toddlers from low SES families seemed to be protected against the possible detrimental influence of inconsistent/coercive parenting on IC. Looking to our data, these two environmental factors (SES and parenting) interact with children’s temperamental predispositions (particularly with EC) to explain both resilience and vulnerability in the face of unfavourable environmental conditions in relation to early individual differences in IC. Expanding on this idea, EC might be considered as a protective factor for children raised in disadvantageous environments as early as from toddlerhood. In line with this, higher levels of EC have been observed to prevent the development of externalizing behaviour problems in three-year-olds who present higher levels of reactivity [65]. Likewise, our results identify toddlers with lower EC as more vulnerable to socioeconomic deprivation and negative parenting practices. These factors, when combined with disadvantaged economic circumstances, may represent a cumulative risk effect. However, more research is still required to determine to what extent certain temperament profiles constitute a risk to the development of cognitive skills such as IC in addition to the characteristics of the environment [74]. An alternative perspective is to consider that children with certain temperamental characteristics can be more susceptible to the influence of environmental factors, as is proposed by some authors [75]. Further research is also needed in order to explore whether the relationship among variables varies across the development and the explanatory mechanism underlying such associations. 

Some limitations should be noted when considering the results of the present study. The sample size in the current study was relatively small for this kind of research. Increasing the sample size would allow testing more complex multidimensional models to better understand the interrelation between environmental variables and children’s temperamental profiles, enlightening our understanding of individual differences in the development of cognitive abilities such as IC. Besides, we only considered a general distal measure regarding the socioeconomic characteristics of the environment. The SES index is a broad measure including parents’ education, occupation, and family incomes. However, it has been proposed that household features would be not only a more proximal measure, but also an important aspect to consider in order to have a complete picture of the characteristics of the context in which children are raised. Research on the topic has revealed that household chaos and SES can contribute separately to explain individual differences in cognitive development [30,76]. There is also some evidence indicating that home chaos could be mediating the impact of SES on children’s cognitive development [77]. Another variable not measured in this study that could be mediating the influence of SES on cognitive growth is nutrition [78,79]. In relation to measuring parenting, we asked parents about the parenting practices that in general both caregivers usually applied with their children, but an independent measure of parenting was not obtained for each caregiver. Future research may additionally explore the independent influence of father and mother parenting practices. There is some evidence indicating that the father and mother may independently contribute to children’s cognitive development in a significant way [37,80]. This might also be relevant to understand individual differences in the development of IC and other self-regulatory skills. Additionally, parenting behaviour was assessed through parent report. Some studies have signalled that self-report measures of parenting practices and observational measures of parenting behaviour in parent–child interaction settings should be jointly considered to increase the reliability of the parenting style assessment and obtain richer information about parenting practices [81]. In fact, a recent systematic review of the literature concluded that the robustness of the results may increase when direct observational measures of parenting are obtained [42]. Apart from that, our study only included a single measure of IC. We used a specific delay task for measuring the ability of toddlers to inhibit their behaviour when they are required to self-regulate. Delay tasks are widely used in the field of developmental psychology to study individual differences in IC and self-regulation during childhood. A variety of delay tasks have been developed, involving different kinds of situations in which children are asked to control themselves. Therefore, it is possible to design future studies including multiple different delay tasks. This will benefit the robustness of the measure, accounting for the consistency of children’s responses across different situations. The use of batteries of tasks for measuring is an extended practice in the study of cognitive development, especially with young children [23,40,82,83], when situational factors may impact the reliability of the measurement more easily. Finally, we observed the impact of environmental factors within a very specific time window. Thus, it is uncertain whether the time that toddlers were exposed to the environmental conditions additionally contributes to explain individual differences in IC at this age. This has been found to be an important factor to consider when studying developmental cognitive outcomes in older children [84,85]. 

To conclude, several practical implications can be derived from the present research. It is noteworthy that toddlers with poorer EC could be at risk for potential psychosocial problems due to poorer IC skills and self-regulation, particularly when raised in low SES contexts and exposed to negative parenting practices. However, it remains unclear whether the interaction pattern among the studied variables would change over time as children grow older and what would be the effects in the long term. Future studies could expand the current findings by exploring the longitudinal trajectory of IC from infancy to childhood, including the assessment of academic and psychosocial outcomes. This kind of research may help to identify not only individual profiles of children at risk for psychological maladjustment but also characteristics of the context that may have a detrimental effect on the development of IC and self-regulation, as well as potential protective factors. Our findings also revealed that adverse environmental conditions already have an impact in toddlerhood. Taken together, this suggests that the detection of children at risk and the implementation of preventive interventions should start even before the age of two in order to address possible disparities in early development and prevent later negative outcomes related to impaired IC skills, such as learning disabilities, attention disorders or externalising behaviour problems. Some authors propose that the sooner we intervene, the greater impact on the development of cognitive abilities [86]. There are a variety of approaches in the literature addressing different forms of intervention in order to palliate the effects of disadvantageous environmental conditions on children’s cognitive development. Some investigations focused on improving cognitive abilities through cognitive training [87,88], while others advocate for parent-centred interventions [89,90,91] or simply by improving families’ economic conditions [92,93]. A significant portion of these investigations find positive effects in reducing the cognitive gap between children from different SES backgrounds. Our research notably stresses the importance of educating caregivers on parenting practices that promote self-regulation in children. According to our data, toddlers raised in low SES contexts whose caregivers tend to be more inconsistent and coercive may have a higher risk for later behaviour problems. However, the interplay among the different environmental factors should also be taken into account. It has been recently proposed that a multi-dimensional approach could be even more effective [94]. Additionally, it has also been suggested that the individualization of intervention, considering initial individual differences of children, may benefit the effect of such intervention [88,95]. This aligns with our findings on the moderation effect of temperamental EC on the impact of environmental factors on toddlers’ individual differences in IC. 

## 5. Conclusions

The present study offers valuable insight into individual differences in an important developmental milestone in toddlerhood: the use of IC abilities to self-regulate is. We found that poorer IC in toddlers was associated with lower SES, inconsistent and coercive parenting, and lower EC. Moreover, we demonstrate that there is an interplay between environmental factors and constitutional factors, highlighting the importance of considering how these influential factors interact in relation to the development of IC in the context of self-regulation. Thus, our study sheds light on how SES, parenting and temperament are impacting toddlers’ IC, but also suggests that the interrelation among these variables influences the pathways to which IC develops in early years. Subsequent research may expand our results through the longitudinal study of the development of IC from infancy. Other aspects that future studies could additionally measure in relation to the emergence of individual differences in IC would be the amount of exposure to adverse socioeconomic conditions, the development of children’s language, attendance to nursery, parent–child interaction, home environment and sources of cognitive stimulation or nutrition. Likewise, our research may inspire future longitudinal research investigating the trajectories of the development of IC and self-regulation in early years by considering the impact of environmental variables in interaction with individual differences of children’s constitutional aspects such as temperament. 

At the same time, our findings contribute knowledge that may help to improve the design of interventions aiming at cushioning the impact of socioeconomic inequalities on children’s cognitive development, specifically with regard to IC and self-regulation. This may lead to more effective interventions that address the problem with a broader multidimensional perspective and take into account individual differences of children in constitutional aspects, such as temperament. In addition, this knowledge could inform policies that intend to reduce disparities among children from diverse SES backgrounds. Thus, studies such as the present one may lead to changes in general policies driven to palliate the effects of poverty in our society by, for example, supporting families with lower economic resources or promoting general educational programmes. This might be particularly relevant for countries with marked socioeconomicinequalities or impoverished regions.

## Figures and Tables

**Table 1:** Descriptive statistics for all the variables included in this study.

	Measures	Valid *n*	Mean	SD
Inhibitory Control	Snack Delay task (raw scores)	56	29.85	10.07
Temperament (raw scores)	ECBQ Surgency/Extraversion	57	5.19	1.03
	ECBQ Negative Affectivity	57	2.20	0.61
	ECBQ Effortful Control	57	4.97	1.07
Parenting (raw scores)	Inconsistency/coercive parenting scale	57	0.63	0.30
	Acceptation/sensitivity parenting scale	57	2.11	0.30
SES	SES index (z score)	59	0.02	0.75
	Parents Occupation (1–9)	59	5.17	1.20
	Parents Education (1–7)	59	5.40	1.90
	Income-to-need ratio	59	1.99	0.93

**Table 2:** Correlation of temperament, SES and parenting measures with the performance of toddlers in the Snack Delay task. The CIs for the correlation coefficients are provided between brackets.

		Snack Delay
Temperament	ECBQ Surgency/Extraversion	−0.07 [−0.32, 0.19]
	ECBQ Negative Affectivity	−0.06 [−0.32, 0.2]
	ECBQ Effortful Control	0.33 * [0.07, 0.54]
Parenting	Coercion/inconsistency	−0.25 * [−0.48, 0.01]
	Acceptation/sensibility	0.13 [−0.14, 0.38]
SES	SES general index	0.37 ** [0.13, 0.57]

* *p* < 0.05; ** *p* < 0.01.

## Data Availability

The data presented in this study are available on request from the corresponding author. The data are not publicly available due to privacy/ethical restrictions.

## Supplementary Materials

The following supporting information can be downloaded at: https://www.mdpi.com/article/10.3390/children10061085/s1, Table S1: Coefficients for the moderation analysis testing the interplay between SES, coercive/inconsistent parenting and IC in the Snack Delay task.
