# Supplementary material for: The Interplay between Socioeconomic Status, Parenting and Temperament Predicts Inhibitory Control at Two Years of Age
Source: Children (Basel). 2023 Jun 20;10(6):1085. doi: 10.3390/children10061085 (PMC10297106; doi:10.3390/children10061085)
Supplement: Supplementary file 1 [file children-10-01085-s001.zip › children-2356552-supplementary.pdf]

Supplementary Table 1

Coefficients for the moderation analysis testing the interplay between SES, coercive/inconsistent parenting and IC in the Snack Delay task. Significance levels: # p<0.1, *p<0.05, **p<0.01. 95% CI are provided between squared brackets.

**DV :Snack Delay performance**	***R^2***	***B***	***t***	***95%CI***	***ΔR^2***
	0.06#				
SES		0.37	1.67#	[-0.08,0.81]	
	0.16*				0.10*
SES		0.43	2.03*	[0.00,0.86]	
Inconsistent /Coercive Parenting		-1.12	-2.27*	[-2.11,-0.13]	
	0.20*				0.03
SES		0.48	2.24*	[0.05,0.91]	
Inconsistent /Coercive Parenting		-1.22	-2.48*	[-2.22,-0.23]	
EC		-0.20	-1.36	[-0.51,0.11]	
	0.29**				0.09*
SES		-0.70	-1.32	[-1.77,0.37]	
Inconsistent /Coercive Parenting		-1.74	-3.38**	[-2.77,-0.70]	
EC		-0.28	-1.9#	[-0.57,0.02]	
SES x Parenting		2.24	2.40*	[0.36,4.13]	
	0.35**				0.06*
SES		2.27	1.37	[-1.09,5.63]	
Inconsistent /Coercive Parenting		-1.41	-2.66**	[-2.48,-0.34]	
EC		0.03	0.16	[-0.41,0.47]	
SES x Parenting		1.81	1.93#	[-0.08,3.7]	
SES x EC		-0.58	-1.88#	[-1.19,0.05]	
	0.35**				0.00
SES		2.05	1.02	[-2.03,6.13]	
Inconsistent /Coercive Parenting		-0.38	-0.07	[-10.81, 10.06]	
EC		0.18	0.24	[-1.35,1.7]	
SES x Parenting		1.87	1.88#	[-0.15,3.89]	
SES x EC		-0.54	-1.45	[-1.28,0.21]	
Parenting x EC		-0.22	-0.20	[-2.41,1.98]	
	0.43**				0.08*
SES		-8.32	-1.69	[-18,31,1.67]	
Inconsistent /Coercive Parenting		-4.32	-0.83	[-14.84,6.2]	
EC		0.09	0.12	[-1.37,1.54]	
SES x Parenting		21.00	2.49*	[3.92,38.08]	
SESXEC		1.64	1.61	[-0.42,3.69]	
Parenting x EC		0.73	0.66	[-1.52,2.98]	
SES x Parenting x EC		-4.06	-2.28*	[-7.66,-46]	
